# Community-University Partnership Characteristics for Translation: Evidence From CDC's Prevention Research Centers

**DOI:** 10.3389/fpubh.2020.00079

**Published:** 2020-03-20

**Authors:** Belinda-Rose Young, Kimberly D. Leeks, Connie L. Bish, Paul Mihas, Rose A. Marcelin, Jennifer Kline, Brigette F. Ulin

**Affiliations:** ^1^Oak Ridge Institute for Science and Education, Oak Ridge, TN, United States; ^2^Department of Health Behavior, The University of North Carolina at Chapel Hill, Chapel Hill, NC, United States; ^3^The Centers for Disease Control and Prevention, Atlanta, GA, United States; ^4^Howard W. Odum Institute for Research in Social Science, The University of North Carolina at Chapel Hill, Chapel Hill, NC, United States; ^5^Association of Schools and Programs of Public Health, Washington, DC, United States; ^6^Aveshka, Vienna, VA, United States; ^7^Tennessee Department of Health, Nashville, TN, United States

**Keywords:** translation, partnerships, community engagement, trust, knowledge to action, partnership development, partnership maintenance

## Abstract

**Background:** The Centers for Disease Control and Prevention's Prevention Research Centers (PRC) Program supports community engagement and partnerships to translate health evidence into practice. Translation is dependent on the quality of partnerships. However, questions remain about the necessary characteristics to develop and maintain translation partnerships.

**Aim:** To identify the characteristics that influence community-university partnerships and examine alignment with the Knowledge to Action (K2A) Framework.

**Methods:** Final Progress Reports (*N* = 37) from PRCs funded from September 2009 to September 2014 were reviewed in 2016–2017 to determine eligibility. Eligible PRCs included those that translated an innovation following the applied research phase (2009–2014) of the PRC award (*n* = 12). The PRCs and the adopters (i.e., community organizations) were recruited and participated in qualitative interviews in 2017.

**Results:** Ten PRCs (83.3% response rate) and four adopters participated. Twelve codes (i.e., elements) were found that impacted partnerships along the translation continuum (e.g., adequate communication, technical assistance). Each element aligned with the K2A Framework at multiple steps within the translation phase. The intersection between the *element* and *step in the translation phase* is termed a “characteristic.” Using interview data, fifty-two unique partnership characteristics for translation were found.

**Discussion and Conclusion:** The results suggest multiple characteristics that impact translation partnerships. The inclusion of these partnership characteristics in policies and practices that seek to move practice-based or research-based evidence into widespread use may impact the receptivity by partners and evidence uptake by communities. Using the K2A Framework to assess translation partnerships was helpful and could be considered in process evaluations to inform translation partnership improvement.

## Impact Statement

**Implications**: Investing time into building sound translation partnerships can lead to increased meaningful engagements, capacity building, and evidence uptake. By applying the Knowledge to Action Framework to our analyses, we were able to assess and describe elements that influence community-university partnerships. This paper introduces a list of partnership characteristics to be considered along the steps of translation.

## Introduction

The goal of *translation* is to move an innovation (e.g., evidence-based research) into widespread use (e.g., practice settings) (1). While the concept of translation seems simple, there are many underlying elements that contribute to the process. This has led to both the exploration and examination of those elements that facilitate the translation of research. Some of the elements that focus on the science of relaying or applying information include: *implementation* (e.g., how to best apply evidence in a real-word setting) (2); *systems thinking* (3, 4); and *dissemination* (e.g., the creation or strengthening of an infrastructure to facilitate the distribution of scientific evidence to end-users) (5). Other elements focus on *who is* involved and/or *how* they are involved, such as: *community engagement* (in which communities are included in addressing community problems) (6), *democratic engagement* i.e., integrating community stakeholders into research as co-generators of knowledge) (7), *the role of “knowledge brokers”* (i.e., mediators that facilitate communication between community stakeholders and academic researchers) (8, 9), *mentoring* (e.g., imparting skills to new scientists to effectively engage community stakeholders, and peer-mentoring for skill-building among professionals in academia and community to effectively engage one another) (10) *and community-university partnerships* (i.e., developing strategic partnerships for the purpose of tackling a community problem through the implementation of research evidence) (11).”

### Prevention Research Centers

The Centers for Disease Control and Prevention (CDC) funds the Prevention Research Centers (PRC) Program, which awards an accredited school of public health or an accredited osteopathy or medical school with a preventive medicine residency program to build and/or maintain infrastructure, via a center, to conduct community-based applied research projects (12). The PRCs are awarded for 5 years to implement an applied research project. Over the course of the last three funding opportunity announcements (FOAs), there has been a gradual transition for PRCs to move beyond accomplishing applied research to establishing a translation plan and in some cases actually beginning the translation of their proven, effective prevention intervention into public health practice with a partner for community-wide benefit. Because each PRC has ongoing partnerships with multiple community organizations, the PRC's data from past funding cycles (e.g., 2009–2014) can be used to examine how community partnerships can be enhanced to support translation efforts before, throughout, and post funding.

### Community-University Partnerships

Community-university partnerships are collaborative relationships between academicians and a community entity (e.g., coalition, health agency) (13) for the purposes of generating new knowledge and/or bridging the gap between knowledge development and application (11). These partnerships also have the potential to enhance translation efforts (11). Community-university partnerships have been shown to mitigate the time lag in evidence uptake (14); increase sensitivity to a community's unique needs and circumstances (15); and increase each partner's exposure to new resources and services (16).

While the concept of community-university partnerships is well-studied, questions still remain about the characteristics needed to guide the relationship through the translation process (11). We operationally define *partnership characteristics* for this paper as *the elements that are essential to support community-university partnerships for translation*. In any given partnership there are those who initiate the translation process (by proposing that an innovation be translated) and those who decide to adopt the innovation. For the purpose of our study, we focused on partnerships between PRCs (the *initiators*) and community organizations (the *adopters*) in the context of translation. These partnerships were established prior to the 2009–2014 PRC Program funding cycle, with the intent of working together to translate evidence into the adopters' communities.

### Knowledge to Action Framework

**Figure 1:**
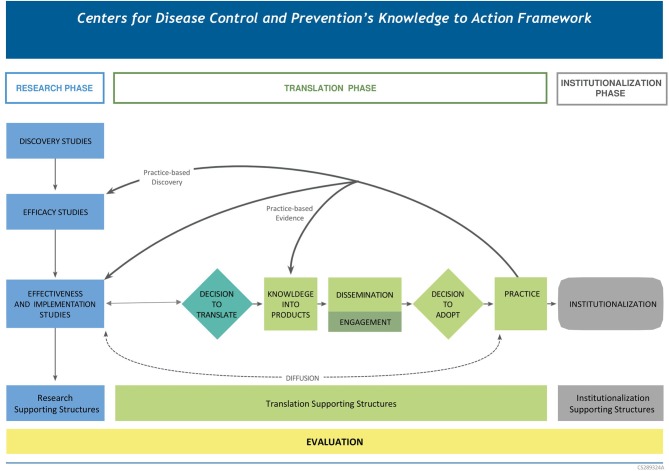
Centers for Disease Control and Prevention Knowledge to Action Framework.

The Knowledge to Action (K2A) Framework is a bi-directional schematic depicting the process for developing, testing, and translating research evidence (from left to right), or for translating practice-based evidence and testing out practice-based discoveries (right to left) (17). The K2A Framework is an organizing framework for translation that is comprised of 3 phases (research, translation, and institutionalization) and 8 iterative steps. The majority (*n* = 6) of the 8 steps are within the translation phase of the framework. The steps within the translation phase, which are needed to translate research into sustainable, widespread practice, include: (1) Decision to translate, (3) Knowledge into Products, (4) Dissemination, (5) Engagement, (6) Decision to adopt, and (7) Practice (Figure 1) (18). We examine the K2A by applying evidence from a sample of PRCs.

The questions guiding this study include:

1. What are the characteristics that influence the development and maintenance of community-university partnerships?
2. How do the partnership characteristics align with the Knowledge to Action Framework translation phase steps?

## Materials and Methods

### Eligibility

The PRCs (i.e., initiators) were eligible to participate if they (1) were funded during the September 30, 2009 to September 29, 2014 funding cycle, and (3) partnered with a community organization (i.e., adopter) to translate an evidence-based innovation (e.g., tool, policy, or intervention) during that funding cycle. The innovation translated could have been developed or adapted by the initiator.

To determine eligibility, the final progress reports (FPRs) from all 2009–2014 PRC Program-funded awardees were reviewed in 2016–2017. The FPRs are documents written by the PRCs and submitted to the CDC PRC Program at the conclusion of the funding cycle. These internal documents include information on the activities, projects, and community organizations that the initiators worked with over the 5 year period. An eligibility check sheet and accompanying search guidance document were created to help the reviewers determine eligibility. While the description of each initiators' projects within their respective FPR was read in totality, the reviewers utilized 16 search terms to assist them. These search terms (Table 1) were informed by the K2A Framework planning guide (18). Eligibility screening was conducted independently by two reviewers (B.-R.Y and R.M.) and arbitrated by two others (K.L. and C.B.). A data abstraction sheet was also created and used by the two reviewers to independently abstract data from the FPRs.

**Table 1:** Key words used when searching FPRs.

Bench to trench Bench to bedside Diffusion (reviewer must determine relevancy) Discovery into action Dissemination (reviewer must determine relevancy) Fidelity Knowledge integration Knowledge to action Knowledge transfer Knowledge translation Research to practice Research use Scaled up/scale Synthesis (of)/research synthesis (for) Shown (to) Translate/translation (and any of its synonyms)

### Recruitment and Interviews

Eligible initiators were the Principal Investigators of the projects, as noted within the FPR. Each eligible initiator was contacted via email in 2017 and asked to participate in a qualitative, semi-structured interview. Recruitment emails provided a description of the study, the objectives, and a copy of the interview consent form. Eligible initiators who agreed to participate were asked to provide an email address for the adopter of the translated innovation. The adopters were sent recruitment emails with the same information noted above. Because the interviews were conducted 3 years post-funding cycle (2017), staff turnover at the community organizations made it challenging to find staff to interview who had been involved in all phases of the innovation's translation.

Qualitative interviews provide a rich source of data allowing for an explanation for an observed phenomena. Tewksbury (19) notes that qualitative interviews provide an “unlimited range of possibilities with accompanying context” (p. 44). Customized semi structured interview guides were developed, for each audience (initiators and adopters), which included questions that aligned with the K2A Framework Translation Phase (Table 2). We used semi-structured interviews where each respondent was asked the same questions in the same sequence. In some cases, the interviewer added a probe or follow-up question for clarity or to generate more detail regarding the topic at hand. Questions regarding the *decision to translate* and *knowledge into products*, were only asked of the initiators; and questions about the *decision to adopt* were only asked of the adopters. For both initiators and adopters, elements from the practice and technical assistance steps were combined to mitigate respondent burden (Table 2).

**Table 2:** Interview questions by K2A Framework Translation phase step.

**K2A Translation phase step**	**Adopter interview question(s)**	**Initiator interview question(s)**
Decision to translate	N/A	At some point, either you or someone else in your organization decided to translate [Project Name]. • Describe why you decided to translate [Project Name]. By *translate*, we mean “active involvement of your organization, and a partner, in implementing a previously tested [project/tool/strategy] in a community
Knowledge into products	N/A	Before you were able to engage stakeholders, and disseminate information about your [project/tool/strategy] you may have developed products to share • Describe any products or tools used to disseminate the [project/tool/strategy]
Dissemination	• Did you receive any products, tools, or written guidance to help with your decision to adopt [the Initiator's intervention/program]? • Did you provide any resources to the Initiator?	• Is this the way that [Adopter] heard about your work? [*If no*] How did your partner [the Adopter], hear about your work?
Engagement	• How did you hear about [the Initiator's intervention/program]?	• In addition to [the Adopter], who else was helpful during the translation process?
Decision to Adopt	• How did you come to the decision to adopt [the Initiator's intervention/program]?	N/A
*Practice/Technical Assistance*	• Were resources needed to implement [the Initiator's intervention/program]? • What technical assistance or guidance was necessary to implement the [intervention/program]?	Describe the level of involvement that [the Adopter] had in the project. [*if there was involvement*] What support or technical assistance did your organization provide to [the Adopter] during the process to translate the [strategy/intervention/project]

The interview guides were iteratively reviewed for clarity and appropriateness, and refined as needed by the study team prior to study implementation. For consistency, interviews were conducted by one member of the study team, and were conducted separately by audience type (i.e., adopters and initiators were not interviewed together). The interviews were approximately 45 min and were audio-recorded (with interviewee permission) through Skype for Business.

### Data Analysis

Transcription was performed using R 3.1.2 (20). The interviews' mp3 files were first converted to wave format using the tuneR package (21). The transcribeR package (22) was then used to transcribe the audio to text.

#### Coding

A codebook, with 12 pre-determined primary codes (i.e., code) based on the main concepts from the K2A Framework, was designed to assist with the coding process. Each code was defined based on its use in the current study and consistently referred to while coding the data (23). After coder training, which included mock coding, comparison of a coder's work to the definitions in the codebook, and refining of code definition, an analysis of the qualitative interview data was conducted to identify common patterns related to translation and the initiators. Specifically, the researchers generated quotation reports on each code and identified patterns and varying degrees of intensity. For example, for the quotes coded to “dissemination,” we discerned that dissemination was evident at various stages of the translation phase (from general mentions of dissemination strategies to discussion of specific efforts made for their project). Numerous quotes were synthesized to capture the primary takeaways and variation within a given code.

Identifying details were deleted from the transcripts before the analysis began. Two members (B.-R.Y and K.L.) of the analysis team identified, labeled, and categorized the codes in each transcript as well as negotiated and finalized the inductive (i.e., emerging) codes. The six additional codes that emerged were defined and agreed upon by the analysis team (based on their specific use in the current study) and included in the codebook. The analysis team met regularly during the analysis to resolve differences in code application and interpretation, and reach agreement on codes and patterns within and across transcripts. Any disagreement in code application was resolved through review of the codebook and, if necessary, the K2A framework guide (18) (which provided additional context). The software program ATLAS.ti 7 was used to help manage the data.

## Results

### Sample

Twelve of the 37 initiators (32.4%) from the PRC Program were eligible (i.e., they translated an innovation during the immediate past funding cycle) for recruitment; ten participated (83.3% response rate). Five of the 10 initiators were able to provide adopter contact information, often citing staff turnover as a reason for not having contact information 3 years post-funding cycle. Of the five available adopters, four agreed to participate. Semi-structured interviews were conducted with 14 stakeholders (initiators *N* = 10; adopters *N* = 4). Table 3 describes participant and project characteristics.

**Table 3:** Participant and project characteristics.

**Participant characteristics**	**Interviewee (*N* = 14) *n* (%)**
**Interviewee type**	
Adopter	4 (28.6)
Initiator	10 (71.4)
**U.S. Census region of respondent**	
Northeast	2 (14.3)
Midwest	3 (21.4)
West	6 (42.9)
South	3 (21.4)
**Adopter interviewee position (*****n*** **=** **4)**	
Director	2 (50)
Staff	2 (50)
Adopter type (*n* = 4)	
Community based organization	3 (75)
Community representative	1 (25)
Initiator interviewee position (*n* = 10)	
Faculty *alone*	1 (10)
Director *alone*	5 (50)
Principal Investigator and Faculty	1 (10)
Director and Principal Investigator	3 (30)
**Initiator setting (*****n*** **=** **10)**	
School of Medicine	1 (10)
School of Public Health	9 (90)
**Project characteristics**	**Project (*****N*** **=** **10)** *n* (%)
**Health topic**	
Physical activity and Nutrition	1 (10)
Physical activity	4 (40)
Substance use	1 (10)
Noise exposure	1 (10)
Positive youth development	1 (10)
Sexual risk behavior	1 (10)
Obesity	1 (10)
**Priority population**	
Racial minority women	2 (20)
Youth and adolescents	5 (50)
Low-income adults	1 (10)
Obese adults	1 (10)
Entire community	1 (10)
**Length of initiator-adopter relationship before project initiation**	
Less than 6 months	3 (30)
6 months to a year	1 (10)
1 year to 4 years	0
Four years or more	6 (60)

### Codes

In total, 18 codes were used in our analysis (Table 4). Certain codes were frequently used between both groups of participants, or disproportionately among one group. For example, although the sample size of the initiators outnumbered the adopters 5:2, the adopters were more likely to discuss the importance of the codes: *adequate communication, respect culture of setting, respect for diversity*, and *trust/mutual respect*.

**Table 4:** Operational definitions for a priori and inductive codes.

**Code**	**Operational definition**
**Pre-determined codes (K2A steps)**	
Decision to translate	The decision to propel an evidence-based intervention, program, practice, and policy into widespread use
Knowledge into products	Any tangible materials (e.g., tools, toolkits, action guides) created for the purposes of disseminating evidence-based knowledge
Dissemination	A purposeful and facilitated process of distributing information and materials to organizations and individuals who can use them to improve health outcomes
Engagement	The active participation and collaboration of stakeholders who can mobilize resources and influence systems to change policies, programs, and practices
Decision to Adopt	The decision at the organizational or community level to implement a previously tested intervention, program, policy, or practice.
Practice	Performing the tangible tasks and action steps to achieve the entity's public health objectives. The process of putting the intervention, program, policy, or practice into place
**Pre-determined codes (K2A main concepts)**	
Adaptation	Additions, deletions, modifications, substitutions, reordering, or other changes to the intervention, program, policy, or practice
Effectiveness	The extent to which the intended effect or benefits that were achieved under optimal conditions are also achieved in real-world settings
Evaluation	A systematic process for an organization to (1) improve and account for public health actions, and (2) obtain information on its activities, its effects, and the effectiveness of its work to improve activities and describe accomplishments
Resources	Any resources needed or used by an entity for the purposes of improving their capacity to accomplish their public health objectives (e.g., training, resources). These resources are not provided by another entity as a form of technical assistance
Stakeholder	Stakeholders include either: (1) partners, who are equitable collaborators in the translation and widespread use of science-based programs, practices and policies; or (2) individuals who are not directly involved (i.e., external) to the initiator project, but who may have an interest or concern in the project
Technical assistance	The formal or informal engagement of an entity to one or more additional entities for the purpose of improving their capacity to accomplish their public health objectives (e.g., training, resources)
**Inductive codes**	
Expertise/skills	Any knowledge, capability, or proficiency needed or desired to carry out a role or duty within the translation or sustainability of the intervention, program, policy, or practice
Roles	Any positions, and their associated duties or activities, needed to facilitate the translation or sustainability of an intervention, program, policy, or practice
Adequate communication	Clear communication about project expectations, including benefits for all involved
Respect culture of setting	Respect and celebrate the culture of the settings within the community organization, geographical community, and academic environment. Acknowledge differences between partners regarding their work setting
Respect for diversity	Respecting differences in behavioral practices, preferences, and opinions
Trust and mutual respect	Taking time to get to know one another, acknowledging each other's strengths, and having a positive attitude about the collaboration

### Partnership Characteristics

*Partnership characteristics* was operationally defined as the summed elements that are essential to support community-university partnerships for translation. Due to space restraints, we describe some of the characteristics for each K2A Translation Phase step. However, the characteristics are fully summarized in Table 5.

**Table 5:** Partnership characteristics for translation.

**Element**	**Element crosswalk with Knowledge to Action Translation phase steps**					
	**Decision to translate**	**Knowledge into products**	**Dissemination**	**Engagement**	**Decision to adopt**	**Practice**
Adaptation	• Consider the feasibility of adaptation for effective translation • Consider the participatory process to adapt the innovation • Be transparent about factors motivating adaptation to stakeholders			• Be transparent about factors motivating adaptation to stakeholders		
	• Engage with prominent leaders within the community to understand what they think the community needs					• Leverage or build on existing community events/ structure
					• Work together to develop adaptation processes for the innovation [e.g., delivery method, setting, and material(s)]	• Work together to develop adaptation processes for the innovation [e.g., delivery method, setting, and material(s)]
Adequate communication						• Create an environment where it is normative to host regular meetings so that everyone has the opportunity to stay informed • Solicit feedback from end-users in the community about the innovation on a routine basis and further refine innovation as needed • Adopters and Initiators should strategize how to thank the community for their involvement at the end of the project and provide them with information on how to maintain the health behavior
Effectiveness	• Before translating, consider if you (or someone else) had positive effects using this innovation in a similar group of people					
	• Consider comparing the new innovation to an ongoing community project to see if there are meaningful differences that were not previously seen				• Consider comparing the new innovation to an ongoing community project to see if there are meaningful differences that were not previously seen	• Consider comparing the new innovation to an ongoing community project to see if there are meaningful differences that were not previously seen
Evaluation	• Consider conducting formative research or social assessment					
					• Begin with evaluation in mind. Discuss what both groups want to achieve, and work together to develop/select an evaluation tool	• Begin with evaluation in mind. Discuss what both groups want to achieve, and work together to develop/select an evaluation tool
Expertise/skills				• Be willing to accept the expertise of your partner. A lack of willingness could hinder the study's effectiveness and reach	• Be willing to accept the expertise of your partner. A lack of willingness could hinder the study's effectiveness and reach	• Be willing to accept the expertise of your partner. A lack of willingness could hinder the study's effectiveness and reach
		Collaboration in product design can lead to an understanding of, and use of, existing expertise/skills among Adopters and Initiators				
					Utilize expertise/skills of all stakeholders to increase the uptake of the innovation	Utilize expertise/skills of all stakeholders to increase the uptake of the innovation
Resources	• Consider all of the human resources that you have available to you. This has implications for design, budget, feasibility, and timeline • Consider research design/setting back-up plans in case staffing and funding changes • Consider budgeting for: national experts in the public health area of interest to help select an evidence-based practice			• Consider all of the human resources that you have available to you. This has implications for design, budget, feasibility, and timeline	• Consider research design/setting back-up plans in case staffing and funding changes • Consider all of the human resources that you have available to you. This has implications for design, budget, feasibility, and timeline Consider research design/setting back-up plans in case staffing and funding changes	• Consider research design/setting back-up plans in case staffing and funding changes • Consider budgeting for: members of the community to fill staffing needs
	• Existing human and financial resources (from stakeholders) can enhance project reach	• Existing human and financial resources (from stakeholders) can enhance project reach	• Existing human and financial resources (from stakeholders) can enhance project reach	• Existing human and financial resources (from stakeholders) can enhance project reach	• Existing human and financial resources (from stakeholders) can enhance project reach	• Existing human and financial resources (from stakeholders) can enhance project reach
	• Leverage existing, in-kind resources to improve reach				• Discuss what types of resources (e.g., equipment) will be purchased for the project and by whom before project initiation	• Discuss what types of resources (e.g., equipment) will be purchased for the project and by whom before project initiation • Seek for multiple funding opportunities for both the Adopting and Initiating entities to apply for, in order to increase availability of resources and partnership longevity
Roles					• Be open to renegotiating roles	• Be open to renegotiating roles
					• Faculty/staff whose job descriptions align with the proposed work may find it easiest to incorporate the responsibilities into their existing work setting	• Faculty/staff whose job descriptions align with the proposed work may find it easiest to incorporate the responsibilities into their existing work setting
					• Collectively decide the roles that everyone will take on. Roles should not be decided before the *decision to adopt* phase. Roles should be explicitly defined (e.g., what does it mean to have a Community PI?), and reviewed periodically to ensure consistent buy-in	• Collectively decide the roles that everyone will take on. Roles should not be decided before the *decision to adopt* phase. Roles should be explicitly defined (e.g., what does it mean to have a Community PI?), and reviewed periodically to ensure consistent buy-in
Stakeholder	• Considering who the end-user might be prior to deciding to translate • [For Initiators who had a prior partnership with the Adopter] Be mindful of prior commitments to continued partnership			• [For Initiators who had a prior partnership with the Adopter] Be mindful of prior commitments to continued partnership		
	• Create a structure that incorporates the local authorities and champions	• Create a structure that incorporates the local authorities and champions	• Create a structure that incorporates the local authorities and champions	• Create a structure that incorporates the local authorities and champions	• Create a structure that incorporates the local authorities and champions	• Create a structure that incorporates the local authorities and champions
	• [For Initiators] Leverage existing networks to build partnerships for adoption; and engage national stakeholders to increase reach • [For Initiators] (if applicable) get buy-in from existing community advisory board before changing health topics	[For Initiators] (if applicable) get buy-in from existing community advisory board before changing health topics	[For Initiators] Leverage existing networks to build partnerships for adoption; and engage national stakeholders to increase reach	• [For Initiators] Leverage existing networks to build partnerships for adoption; and engage national stakeholders to increase reach • [For Initiators] Identify people in the community who understand the concern and work with them to adapt the project • Work with senior staff of the Adopting organization to ensure that project responsibilities are integrated into the staff member's duties • [For Adopters] Feel free to arrange meetings between senior staff of your agency and Initiators to facilitate the uptake of an innovation	• [For Initiators] Identify people in the community who understand the concern and work with them to adapt the project • Work with senior staff of the Adopting organization to ensure that project responsibilities are integrated into the staff member's duties • [For Adopters] Feel free to arrange meetings between senior staff of your agency and Initiators to facilitate the uptake of an innovation • [For Adopters] Inform stakeholders (e.g., community coalition) of project for larger buy-in and resource access	• [For Initiators] Identify people in the community who understand the concern and work with them to adapt the project • Work with senior staff of the Adopting organization to ensure that project responsibilities are integrated into the staff member's duties • [For Adopters] Feel free to arrange meetings between senior staff of your agency and Initiators to facilitate the uptake of an innovation • [For Adopters] Inform stakeholders (e.g., community coalition) of project for larger buy-in and resource access
Respect culture of setting	• Consider if the project (as previously used) was designed and suitable for the new community setting					
				• An environment that is culturally sensitive and relevant (e.g., using the local caterer) builds buy-in and facilitates engagement	• An environment that is culturally sensitive and relevant (e.g., using the local caterer) builds buy-in and facilitates engagement	• An environment that is culturally sensitive and relevant (e.g., using the local caterer) builds buy-in and facilitates engagement
						• Work together to leverage the existing structure of the Adopting organization to better integrate the research into the community
Respect for diversity				• Have an attitude that is sensitive to the culture, practices, and opinions of others. Both Adopters and Initiators bring expertise to the table	• Have an attitude that is sensitive to the culture, practices, and opinions of others. Both Adopters and Initiators bring expertise to the table	• Have an attitude that is sensitive to the culture, practices, and opinions of others. Both Adopters and Initiators bring expertise to the table
				• Create a socio-structural environment that honors the intellect and contribution of either partner	• Create a socio-structural environment that honors the intellect and contribution of either partner	• Create a socio-structural environment that honors the intellect and contribution of either partner
				• Demonstrate cultural humility and engage in participatory decision-making	• Demonstrate cultural humility and engage in participatory decision-making	• Adopters should take the initiative to orient the Initiators to the culture of their community. Likewise, Initiators who work in academia should be transparent about the academic/funding demands • Demonstrate cultural humility and engage in participatory decision-making
Technical assistance		[For Initiators] Conceptualize products (e.g., training manual) that will be useful to engage the Adopter initially and during implementation				[For Initiators] Conceptualize products (e.g., training manual) that will be useful to engage the Adopter initially and during implementation
					• [For Initiators] Create a structure where the Adopting organization has the freedom to lead the implementation (with technical, human, and financial support from you) for project sustainability	• [For Initiators] Create a structure where the Adopting organization has the freedom to lead the implementation (with technical, human, and financial support from you) for project sustainability
						• Ensure proper training of the innovation to those directly involved and to stakeholders not directly involved, but where buy-in is needed • Discuss a process for bi-directional technical assistance (e.g., will we have “check-ins” bi-weekly to see who needs assistance) • Collectively develop reference products/ protocols for the Adopting community at the end of the project (especially if the Adopters/Initiators will not continue the project)
Trust and mutual respect	• [For Initiators] Consider your public image within the community, as it can help/hinder the uptake of your innovation				• While a certain level of rigor is needed to ensure the effectiveness of the translated innovation, there should be trust in the Adopters' wisdom of the community and how it should be approached	• While a certain level of rigor is needed to ensure the effectiveness of the translated innovation, there should be trust in the Adopters' wisdom of the community and how it should be approached
						Create an environment that promptly deals with contention through previously agreed upon guidelines
	• Develop and maintain active engagement with key stakeholders at major decision points	• Develop and maintain active engagement with key stakeholders at major decision points	• Develop and maintain active engagement with key stakeholders at major decision points	• Develop and maintain active engagement with key stakeholders at major decision points	• Develop and maintain active engagement with key stakeholders at major decision points	• Develop and maintain active engagement with key stakeholders at major decision points

### Decision to Translate

Initiators noted several reasons for deciding to translate a particular innovation, such as familiarity with the innovation, change in funding level, interest in testing generalizability, the innovation's prior success rate, or because the adopter requested the innovation. In addition, some initiators mentioned that there was a desire to help the community-stakeholders because they did not have access to health promotion resources and because, as initiators, they had learned lessons from previous attempts to implement and could apply that knowledge here.

“We wanted to do that because we saw a great need there. We knew that [the stakeholders] had less access to health promotion, and we'd learned over the course of that [prior] study that they had a lot less help available to them. And then what we tried to do our first time out was just try to deliver [the innovation] as originally developed…and we learned a very hard way that it wasn't a good fit. And it was clear that it would need to be adapted in order to meet their needs.” –Initiator

During the process of deciding to translate, some initiators mentioned that there were internal conversations about the feasibility of translation, and that formative assessments were conducted to understand the community's needs and the level of receptivity to an innovation.

“We spent two years doing that and so that's kind of the formative part and we found that the [stakeholders] were very, very receptive. They got it. They understood the idea. They saw the relevance to their own communities. So we wrote another research proposal for [it].”- Initiator

Initiators funded through the PRC Program must have a community advisory board (CAB) comprised of members from local and surrounding communities. In some cases, members of an initiator's CAB were involved in the decision of whether or not to translate the innovation with an adopter.

Well the [adopter], they had a program, we kind of tested it a little bit. It needed a lot of refining. Everybody wanted to develop something yesterday. So they asked us if we would be willing to work with them. Well, having a very community engaged stakeholder-driven strategy, we couldn't agree to do that until we went to our partners and asked them how they felt about that - was it OK to go from [our state] to a national focus?” -Initiator

### Knowledge Into Products

Initiators described the process for developing products for dissemination. While most initiators noted the development of toolkits and curricula, one initiator discussed the development of media-oriented products. These media products aimed to create interest in the project throughout the entire community, not just community-stakeholders.

“…it was what we call agenda setting, which is using local media to kind of raise awareness and generate excitement. And that consisted of newspapers about local opinion leaders and highly visible [stakeholders]. That came out in their community newspapers, radio interviews on their radio stations, multimedia presentations on their websites and their Facebook page.” - Initiator

### Dissemination

The initiators discussed dissemination efforts at various stages of the translational phase; either describing dissemination strategies generally, specific efforts made to the project, or in relation to the scope of the dissemination. One initiator mentioned that the time spent planning the scope of their dissemination efforts was made at the very beginning.

“But in terms of dissemination of the program it was a national dissemination effort. The translation and dissemination process was part of the planning from the beginning.” -Initiator

Initiators also described how the adopter was informed about their innovation. One initiator describes how they met their adopter, who operates in a different state.

“They heard about it from the conference. So it was pretty much by all word of mouth; relational, was all relational communication, and pretty much everything in [that community] is that way. It's all who knows who and who's involved in this. And so it was friends of friends of friends, and or somebody heard something at a talk and went home and told [a stakeholder] ‘hey we need this.”’ – Initiator

### Engagement

Initiators who had preexisting relationships with the adopter described a commitment to their partners.

“We chose those three sites because they had already been our partners. We already had a commitment with them that they would be a part of the grant. We never considered going with anyone else, because they were our partners.” -Initiator

Initiators who did not have preexisting relationships with the adopter described how they utilized existing connections to engage potential adopters. The existing relationships enabled buy-in from the community and the adopter.

“We were invited into those communities by people from those communities, which really helped us. It laid a relational foundation.” -Initiator

Adopters who had preexisting relationships with an initiator also discussed their engagement efforts with the community. Speaking on the topic of mobilizing community members, one adopter stated:

“Really, we needed a lot of other community partners to even get our foot in the door. Once we had them, they helped [with adaptation] so that it was appropriate for the community.” - Adopter

### Decision to Adopt

In their decision to adopt, the adopters had to choose whether or not the innovation would be a good fit for their community. One adopter, who did not have a preexisting partnership with the initiator, discussed how they initiated the relationship.

“And so I arranged a meeting for my supervisor to meet with [Initiator] and his associates. And anyway that's how they came here, I wanted them to come here. It's easier to prevent something than try to fix it after it's broken” -Adopter

Initiators also reflected on the adoption experience. Notably, some initiators indicated that they believed the adopter's socio-structural environment played a role in whether or not the adopter chose to participate in the translation efforts. In the following quote, an initiator discusses the intersection of the adopter's *place of employment* and their *job description* to create the socio-structural environment.

“I would say that they were the gatekeepers of the idea of community-based work. I'm sure they probably talked with their employers to make sure that things were okay. It was their roles to be doing this kind of work. It fit in with what they wanted to be doing. It already fit in their job description and they were in positions of influence where they didn't have to sell it to other people.” -Initiator

### Practice/Technical Assistance

Adopters and initiators recounted the practice (i.e., implementation) portion of the translation phase. While the majority of participants believed that the innovation was implemented well, some indicated that the lack of training and/or communication posed problems for implementation.

“The people that were implementing it – my outreach coordinator, my program director, and CAB members should have had some kind of hands on training I believe, in order to be able to better utilize the tool. We would have had a greater advantage of reaching out to the community.” -Adopter

Adopters and initiators discussed how the innovation was implemented from varying perspectives. Overwhelmingly, both initiators and adopters discussed the need for additional stakeholder involvement, who could identify how the innovation should be adapted, for successful implementation.

“The next step in the process was to identify people in the community who understood the concern from their own personal experience … and were willing to come alongside us and direct, basically, direct everything we did from the design of an adaptation of the interventions, to how we deliver them, to what [implementation site to choose].” -Initiator

### Intersection of Partnership Characteristics With K2A Step

All data were condensed into codes. These codes were used to create a compendium of translation partnership characteristics, according to the intersection of code and K2A Framework Translation phase step (Table 5). Specifically, all data (i.e., quotes, responses) were separated into groupings according to each K2A step (Table 2). Within each grouping, data were further separated by code (thus creating subgroupings, or intersections of data between K2A step and code). All quotes within each subgroup were individually reviewed to assess their meaning (as a characteristic). Thus, a *characteristic* is data (i.e., evidence) gathered from participants that characterizes the intersection of a K2A step and a code. The characteristic (e.g., “be transparent about factors motivating adaptation to stakeholders”) could have been mentioned in the context of multiple intersections (e.g., adaptation and *decision to translate*; adaptation and *engagement*).

The findings provide some key characteristics that can be used to guide translation partnerships. While most of the characteristics are applicable to initiators and adopters, some are very specific toward one or the other. Table 5 depicts where each code aligns with the K2A Framework and the associated partnership characteristic.

## Discussion

The K2A Framework was a helpful tool in organizing elements that can impact a partnership along the translation phase. The process of translation requires involvement of both the initiator and the adopter (24). While translation is most often thought to be between community and university partners, it can also occur between two community partners (e.g., health department as initiator and community organization as adopter). Our interview items were inclusive and allowed us to gather information from the *initiator* and *adopter* perspective. The inclusion of both audiences helped us identify characteristics to consider when developing and maintaining partnerships for the purpose of translating applied public health research to practice.

Fifty-two unique partnership characteristics were identified; of which 30 characteristics were relevant to more than one K2A step for a particular element. For example, one of the partnership characteristics that intersects the element *effectiveness* and K2A step *decision to translate* (“Consider comparing the new innovation to an ongoing community project to see if there are meaningful differences that were not previously seen”) is also found at the intersection between *effectiveness* and *decision to adopt*, and again at *effectiveness* and *practice*. An unexpected find, was that some of the participant characteristics were solely relevant to one group vs. the other (e.g., adopter or initiator). This can have grave implications, as a lack of attention to the participant characteristic by that group could hinder progress in both the partnership development and translation project. These group-specific participant characteristics were relevant to the *stakeholder, technical assistance*, and *trust and mutual respect* elements alone.

As discussed in the background section, prior research has examined a singular component of translation (e.g., dissemination), and community-university partnerships in the context of basic research. Our study bridges the gap between community-university partnerships and translation, *and* includes the entire translation pathway. Through this we were able to study critical characteristics of a partnership that should be considered at a specific timepoint in the translation process. Thus, our resulting table of partnership characteristics can be used as a practical guide for both initiators and adopters (Table 5). It includes characteristics specific to each step of translation, which can be used as an organizational standard of practice when engaging with external partners; incorporated into written agreements; and serve as a tool for those who are seeking to build translation partnerships. Indeed, the partnership experience can impact the quality of the translation process and the results.

### Additional Findings

Separately, our findings revealed that half of the initiators included adopters in the decision to translate, therefore yielding an overlap of the codes *decision to translate* and *decision to adopt*. The traditional, linear process of the academician initiating and community member responding was not followed; rather initiators mostly had ongoing community partnerships through Community Advisory Boards or other organizations.

The findings also revealed that several of the inductive codes aligned with the *Interactive and Contextual Model of Collaboration* (ICMC) and were defined accordingly (25). Specifically, the codes were: *trust and mutual respect, adequate communication, respect for diversity*, and *respect culture of setting*. The adopters disproportionately spoke more about *adequate communication, respect culture of setting, respect for diversity*, and *trust/mutual respect*. The frequency of communication around these particular codes speaks to their importance among adopters. Therefore, there is a need for initiators to be sensitive to the existing structures and settings within an adopter's organization when considering translation (e.g., being culturally sensitive, incorporating local resources, solicit feedback from stakeholders). Adopters who discussed these four codes from the ICMC positively did so when initiators allowed the community organizations to drive the adaptation and implementation processes.

### Strengths

There were many strengths associated with this study. Firstly, initiators and adopters were both engaged. This approach provided an opportunity for both sides to freely reflect on the process, and share their lessons learned. Secondly, we inquired about the entire translation phase; not just a single stage. Each stage contains a unique process and understanding, therefore we were able to obtain a holistic view of translation. We were also able to develop a compilation of partnership characteristics that is driven by the data from our study, which creates a more meaningful framework for future endeavors.

### Limitations

While there were many strengths to the study, there were also limitations, most notably the small sample size. With a small population size (*N* = 37) and smaller eligibility pool (*n* = 12), our findings may not be generalizable across all community-based translation partnerships. However, they could be meaningful to groups with similar structures. Second, initiators were asked to participate in a study conducted by their funders which may have elicited some level of social desirability. Thirdly, as described in our methods, we did not ask adopters questions concerning the *decision to translate* or *knowledge into products* based on definitions employed by the K2A. Yet, we found that half of our initiators included adopters in the decision to translate. Thus, there may have been missed data from not asking the adopters the *dissemination, engagement, or decision to adopt* questions (Table 2). Lastly, given that the interviews were conducted 3 years after the conclusion of the funding cycle, respondents may not have recalled all of the elements that impacted the partnership at each step of the translation phase.

## Conclusion and Implications

As co-generators of knowledge, it is imperative to acknowledge and holistically understand the respective environments that both adopters and initiators operate within. Through understanding the community environment (e.g., structure), initiators will be able to appropriately work with adopters to adapt the innovation (26), and navigate established systems of a given community. Likewise, in circumstances where the initiator is the one funded, adopters may gain knowledge in navigating funding cycles and working around specified time constraints. Facilitating an understanding of these critical pieces is imperative in developing effective partnerships for translation. When navigating situations such as knowledge generation, power diffusion, and cultural shifts, the partnership characteristics has the potential to help groups mutually transform their environments. In this paper we examined how, through the lens of the K2A Framework, environmental and behavioral elements collectively contribute to partnerships at multiple stages of translation.

Findings from this study have implications for policy and practice. The partnership characteristics can be integrated into agency, state, or local procedure or policy documents to inform and/or govern engagement in partnerships for translation, and provide an evaluative checklist of those partnerships. Indeed, these characteristics can be used as a deciding factor for whether organizations should expend time and talent with a potential collaborator. Further, it can assist initiators in navigating existing and potential community-university dynamics and partnership needs. Therefore, the partnership characteristics could be used as a mutual agreement to keep all parties accountable. Such accountability can greatly improve relations and transparency with partners, assist in goal setting and timeline development for the project and the organization, and increase productivity. The notions of “stronger together” and “more hands make the work light” are possible when clarity and structure are incorporated. Incorporating these data-driven partnership characteristics into policies and practice can assist in creating a structure that could allow for a high standard of operation.

## Data Availability Statement

The datasets generated for this study are available on request to the corresponding author.

## Ethics Statement

The studies involving human participants were reviewed and approved by Centers for Disease Control and Prevention's Institutional Review Board. The ethics committee waived the requirement of written informed consent for participation.

## Author Contributions

B-RY and KL contributed to the design of the study, data acquisition, data interpretation, manuscript development and revisions, and approved the final version of the submitted manuscript. CB and RM contributed to the design of the study, data interpretation, manuscript development and revisions, and approved the final version of the submitted manuscript. PM and JK contributed to data interpretation, manuscript revisions, and approved the final version of the submitted manuscript. BU approved the final version of the submitted manuscript.

### Conflict of Interest

RM was employed by the company Aveshka subsequent to data collection. The remaining authors declare that the research was conducted in the absence of any commercial or financial relationships that could be construed as a potential conflict of interest.

## References

[1] Sogolow E Sleet D Saul J Dissemination, implementation, and widespread use of injury prevention interventions. In Doll LS Bonzo SE Mercy JA Sleet DA, editors. Handbook of Injury and Violence Prevention. New York, NY: Springer Science+Business Media (2007). pp. 493–510.

[2] Brownson RC Colditz GA Proctor EK Dissemination and Implementation Research in Health: Translating Science Into Practice. New York, NY: Oxford University Press (2017).

[3] BeLue R Carmack C Myers KR Weinreb-Welch L Lengerich EJ. Systems thinking tools as applied to community-based participatory research: a case study. Health Educ Behav. (2012) 39:745–51. 10.1177/1090198111430708 22467637

[4] Kitson AL. The need for systems change: reflections on knowledge translation and organizational change. J Adv Nurs. (2008) 65:217–28. 10.1111/j.1365-2648.2008.04864.x 19032518

[5] Kerner J Rimer B Emmons K. Dissemination research and research dissemination: how can we close the gap. Health Psychol. (2005) 24:443–6. 10.1037/0278-6133.24.5.443 16162037

[6] Ahmed SM Palermo A-G. Community engagement in research: frameworks for education and peer review. Am J Public Health. (2010) 100:1380–7. 10.2105/AJPH.2009.178137 20558798 PMC2901283

[7] Dostilio LD Democratically-engaged community-university partnerships: reciprocal determinants of democratically oriented roles and processes. J High Educ Outreach Engage. (2014) 18:235–244.

[8] Dobbins M Robeson P Ciliska D Hanna S Cameron R O'Mara L. A description of a knowledge broker role implemented as part of a randomized controlled trial evaluating three knowledge translation strategies. Implement Sci. (2009) 4:23. 10.1186/1748-5908-4-23 19397820 PMC2680804

[9] Ward V House A Hammer S. Knowledge brokering: the missing link in the evidence to action chain. Evid Policy. (2009) 5:267–93. 10.1332/174426409X463811 21258626 PMC3024540

[10] Young B-R Williamson HJ Burton DL Massey OT Levin BL Baldwin JA. Challenges and benefits in designing and implementing a team-based research mentorship experience in translational research. Pedagogy Health Promot. (2015) 1:233–46. 10.1177/2373379915600174 26949735 PMC4774555

[11] Williamson HJ Young B-R Murray N Burton DL Levin BL Massey OT. Community-university partnerships for research and practice: application of an interactive and contextual model of collaboration. J High Educ Outreach Engage. (2016) 20:55–84. 28184179 PMC5295659

[12] Centers for Disease Control and Prevention Prevention Research Centers. (2017). Available online at: https://www.cdc.gov/prc

[13] Chapman SA Community-university partnerships: a case study. In Higginbottom G Liamputtong P, editors. Participatory Qualitative Research Methodologies in Health. London: SAGE Publications (2015). pp. 200–1.

[14] Green LW. Making research relevant: if it is an evidence-based practice, where's the practice-based evidence? Fam Pract. (2008) 25(Suppl. 1):i20–4. 10.1093/fampra/cmn055 18794201

[15] Green LW Mercer SL. Can public health researchers and agencies reconcile the push from funding bodies and the pull from communities? Am J Public Health. (2001) 91:1926–9. 10.2105/AJPH.91.12.1926 11726367 PMC1446906

[16] Mersky JP Topitzes J Blair K Translating evidence-based treatments into child welfare services through community-university partnerships: a case example of parent-child interaction therapy. Child Youth Serv Rev. (2017) 82:427–33. 10.1016/j.childyouth.2017.10.002

[17] Wilson KM Brady TJ Lesesne C on behalf of the, NCCDPHP Work Group on Translation An organizing framework for translation in public health: the Knowledge to Action Framework. Prev Chronic Dis. (2011) 8:A46. 21324260 PMC3073439

[18] Centers for Disease Control and Prevention Applying the Knowledge to Action (K2A) Framework: Questions to Guide Planning. Atlanta, GA: Centers for Disease Control and Prevention, US Department of Health and Human Services (2014).

[19] Tewksbury R Qualitative versus quantitative methods: understanding why qualitative methods are superior for criminology and criminal justice. J Theor Philos Criminol. (2009) 1:38–58.

[20] R Core Team. The R Project for Statistical Computing. (2014). Retrieved from: http://www.R-project.org/

[21] Ligges U Krey S Mersmann O Schnackenberg S tuneR: Analysis of Music. (2013). Retrieved from: http://r-forge.r-project.org/projects/tuneR/

[22] Lucas C Knox D Tingley D Scanlan T Sunil S May M transcribeR: Automated Transcription of Audio Files Through the HP IDOL API. (2015). R package version 0.0.0. Retrieved from: http://CRAN.R-project.org/package=transcribeR (accessed June, 2017).

[23] Miles M Huberman A Saldaña J Qualitative Data Analysis: A Methods Source Book. Thousand Oaks, CA: SAGE Publications, Inc (2019).

[24] Kitson AL Rycroft-Malone L Harvey G McCormack B Seers K Titchen A. Evaluating the successful implementation of evidence into practice using the PARiHS framework: theoretical and practical challenges. Implement Sci. (2008) 3:1. 10.1186/1748-5908-3-1 18179688 PMC2235887

[25] Suarez-Balcazar Y Harper GW Lewis R. An interactive and contextual model of community-university collaborations for research and action. Health Educ Behav. (2005) 32:84–101. 10.1177/1090198104269512 15642756

[26] Katz DL Murimi M Gonzalez A Njike V Green LW. From controlled trial to community adoption: the multisite community trial. Am J Public Health. (2011) 101:e17–27. 10.2105/AJPH.2010.300104 21680935 PMC3134505

